# Editorial: Advances in Health-Care Transition for Patients With Childhood-Onset Chronic Diseases: International Perspectives

**DOI:** 10.3389/fped.2018.00080

**Published:** 2018-03-29

**Authors:** Yuko Ishizaki, Mitsue Maru, Hirohiko Higashino

**Affiliations:** ^1^Department of Pediatrics, Kansai Medical University, Moriguchi, Japan; ^2^International Nursing Development, School of Nursing and Rehabilitation, Konan Women’s University, Kobe, Japan; ^3^Higashino Clinic, Higashi-Osaka, Japan

**Keywords:** health-care transition, childhood-onset chronic diseases, pediatrics, adolescent, childhood-cancer, psychology, Turner syndrome

**Editorial on the Research Topic**

**Advances in Health-Care Transition for Patients With Childhood-Onset Chronic Diseases: International Perspectives**

For adult patients with childhood-onset chronic diseases (APCCD), the transition from child- to adult-centered health care is an important topic in both medical practice and economics worldwide. Advances in pediatrics and neonatal medicine have dramatically improved the prognosis for children with previously fatal chronic diseases, allowing them to survive into adulthood (1–3). The number of adolescents undergoing health-care transition is increasing (2–10); therefore, suitable programs are required to integrate the patients into adult-centered care and to help them grow socially and become independent, working adults.

This transition has been defined as a multifaceted, active process that attends to the medical, psychosocial, and educational needs of adolescents as they move from child- to adult-centered care (4). Transition programs should involve patients, families, pediatricians, nurses, adult health-care providers, and other health-care professionals (11, 12), and they should encompass medical, psychosocial, and ethical issues adequate to the various cultural and religious backgrounds.

By presenting a new movement of transition in Asia, Ishizaki et al., integrated the most recent advances in Japan. In terms of APCCD health-care transition history, Japan falls behind the American and European countries. The term “transition program” was introduced in 2006 in the clinical context, and the concept of health-care transition rapidly spread over the last 10 years due to the measures taken by the Japan Pediatric Society and Ministry of Health, Labor, and Welfare.

Various articles from several countries were published, which focused on the health-care transition of patients of childhood cancer. Magni et al. reported their trials named “The Youth Project” in Milano, which supported adults and adolescents (AYA) with pediatric cancer. The project contained not only medical procedures but also creative activities such as fashion collections, song writing, novel writin g, a photography course, and exhibitions and other support activities such as sports.

Using a focus group methodology, Aldiss et al. investigated the views of the professionals involved in transitional care in London, gathering information on the process of transition and its barriers and facilitators. They found eight key factors that impact transition.

Raz et al. evaluated the effect of providing information on the quality of life or mental pain at the time of diagnosis and found that there were differences between younger patients with childhood cancer and the older adults, when they received the diagnosis with or without information about their cancer.

Cancer pain consists of both somatic components and psychosocial elements, affecting the children’s psychosocial development. Scarponi and Pession expressed their opinion about implementing group psychotherapy as a means to get over the pain caused by childhood cancer.

Sakakibara advocated that women with Turner syndrome (TS) received inadequate health care in terms of hormone replacement therapy, to acquire secondary sexual characteristics when patients with TS are managed in only pediatrics. Gynecologists should be involved in the initiation of HRT to create a smoother transition.

These research topics present the latest information about advances in health-care transition obtained from multifaceted professionals. These topics provide useful information for the next steps in promoting health-care transition worldwide.

## Author Contributions

All authors participated in the design of this Research Topic. YI and MM recommended contributors. HH have made an intellectual contribution to the work. All authors approved it for publication.

## Conflict of Interest Statement

The authors declare that the research was conducted in the absence of any commercial or financial relationships that could be construed as a potential conflict of interest.
